# Predictors of Blood Transfusion Following Cesarean Delivery in Resource-Constrained Settings: The Impact of Out-of-Hours Surgery

**DOI:** 10.3390/jcm15218646

**Published:** 2026-11-06

**Authors:** Cezara Muresan, Marius Biriș, Răzvan Roșca, Andrei Pescaru, Cosmin Cîtu, Jasmina Chiriac, Denis Mihai Șerban, Răzvan Betea, Sebastian Ciurescu, Victor Bogdan Buciu

**Affiliations:** 1 Department of Obstetrics and Gynecology, “Victor Babeș” University of Medicine and Pharmacy Timișoara, Eftimie Murgu Square No. 2, 300041 Timișoara, Romania; 2 Clinic of Obstetrics and Gynecology, County Emergency Clinical Hospital “Pius Brînzeu”, 300723 Timișoara, Romania; 3 Department for the Prevention and Control of Healthcare-Associated Infections (CPIAAM), Clinical Hospital of Infectious Diseases and Pulmonology “Dr. Victor Babeș” Timișoara, Gheorghe Adam Street No. 13, 300310 Timișoara, Romania; 4 Doctoral School, “Victor Babeș” University of Medicine and Pharmacy Timișoara, Eftimie Murgu Square No. 2, 300041 Timișoara, Romania; 5 Obstetrics and Gynecology Clinic I, Timișoara Municipal Emergency Clinical Hospital, 300079 Timișoara, Romania; 6 Department of Obstetrics and Gynecology, Municipal Hospital Deva, 330084 Deva, Romania; 7 Obstetrics-Gynecology Clinic IV, Timișoara Municipal Emergency Clinical Hospital, 300079 Timișoara, Romania

**Keywords:** cesarean delivery, blood transfusion, out-of-hours surgery, postpartum hemorrhage, placenta previa, placenta accreta spectrum, maternal morbidity, resource-constrained healthcare

## Abstract

**Background/Objectives:** Perioperative red blood cell (RBC) transfusion after cesarean delivery reflects both obstetric hemorrhagic risk and the capacity of the care environment. We evaluated whether out-of-hours cesarean delivery was independently associated with transfusion in a Romanian tertiary maternity unit operating under constrained resources. **Methods:** We conducted a retrospective unmatched case–control study of cesarean deliveries performed from 2023 to 2025. All eligible transfused women were included as cases; two non-transfused controls per case were randomly selected within each calendar year. Eligibility required preoperative hemoglobin ≥ 10 g/dL and normal coagulation. The primary exposure was out-of-hours surgery, and multivariable logistic regression adjusted for maternal, obstetric, placental, and hematological factors. **Results:** The source population contained 6773 cesarean deliveries, with 608 transfusions (9.0%). The analytical sample comprised 401 cases and 802 controls. Out-of-hours procedures occurred in 64.1% of cases and 52.5% of controls (crude OR 1.62, 95% CI 1.26–2.07). After adjustment, out-of-hours delivery remained associated with transfusion (aOR 2.03, 95% CI 1.45–2.84). Placenta previa, suspected placenta accreta spectrum, and lower preoperative hemoglobin were the strongest clinical predictors. When postoperative/pre-transfusion hemoglobin was added, the out-of-hours association attenuated to an aOR of 1.02 (95% CI 0.63–1.65); sensitivity analyses remained consistent with the primary model. **Conclusions:** Out-of-hours cesarean delivery was associated with approximately two-fold higher transfusion odds after adjustment for the measured clinical factors. Because cesarean indication, urgency, and labor characteristics were not available, this association may partly reflect the different case mix and acuity of out-of-hours surgery rather than the working period itself. Attenuation after adjustment for post-exposure hemoglobin is compatible with, but does not establish greater perioperative blood loss. In high-cesarean, resource-constrained settings, standardized hemorrhage-risk identification and equivalent preparedness across duty periods warrant prospective evaluation in studies that also record indication and urgency.

## 1. Introduction

Cesarean delivery is one of the most frequently performed surgical procedures worldwide and, although generally safe, remains associated with clinically relevant maternal hemorrhage and transfusion requirements. The need for perioperative red blood cell transfusion is strongly influenced by the maternal hematological reserve and obstetric pathology. A systematic review and meta-analysis including 22 studies and 426,094 cesarean deliveries identified placenta previa (OR 9.54), placental abruption (OR 6.77), preterm labor (OR 3.34), unbooked pregnancy (OR 2.42), hypertensive disorders (OR 1.81), and multiple gestation (OR 1.60) as major transfusion-associated factors [1]. Preoperative hematological status is equally important. In a recent Romanian cohort of 932 pregnant women, transfusion occurred in 5.2%, while each 1 g/dL increase in preoperative hemoglobin was associated with a 78% reduction in transfusion odds (OR 0.216); an Hb threshold of 10.9 g/dL yielded an AUC of 0.875 for transfusion prediction [2]. Large contemporary cohorts similarly demonstrate a close association between antepartum anemia/red-cell mass and post-cesarean transfusion [3].

Beyond patient-related risk, increasing attention has been directed toward when obstetric care is delivered. Obstetrics requires uninterrupted 24-h coverage, but personnel availability, workload, senior supervision, and access to supporting services may differ outside routine working hours. Evidence for an “out-of-hours effect” is inconsistent. In a Korean population-based cohort of 90,072 deliveries, severe maternal morbidity was increased during weekday nights (RR 1.26) and weekends (RR 1.58) [4]. A subsequent nationwide analysis of more than 3 million deliveries found approximately 40% higher severe maternal morbidity during nighttime delivery, with larger effects in smaller hospitals and smaller cities [5]. Similarly, analysis of more than 3 million low-risk U.S. births found a modest but consistent weekend effect on maternal and neonatal outcomes (OR 1.13) [6]. Conversely, other U.S. data found no independent increase in severe maternal morbidity during off-hours, and a European tertiary unit with continuous 24/7 presence of experienced staff reported no relevant day–night differences [7,8]. These findings suggest that organizational context, rather than clock time itself, may determine the magnitude of any out-of-hours effect.

This question may be particularly relevant in healthcare environments with limited personnel and resources. The duration of clinical duty may also contribute: Aiken et al. found that major blood loss and adverse neonatal outcomes varied according to the number of hours already worked, with the highest risk occurring after approximately 9–10 h of duty [9]. Such effects may become more clinically relevant when out-of-hours teams are smaller or have reduced immediate access to additional personnel and services.

Romania provides a relevant setting in which to investigate this interaction. A nationwide analysis of 149,466 deliveries from all 191 Romanian hospitals reporting births in 2020 found a cesarean delivery rate of 52.9%, including 49.7% in public and 79.8% in private institutions [10]. International surveillance subsequently estimated the national rate at 51.4% in 2023, which is among the highest reported internationally [11]. The WHO does not recommend applying a fixed cesarean-rate target to individual hospitals, particularly tertiary referral centers with complex case mixes; nevertheless, repeated cesarean exposure progressively changes the obstetric population returning for subsequent pregnancies [12]. In the 2024 PACCRETA population-based study, PAS prevalence among women with a previous cesarean and low-lying/previa placenta ranged from 5% with one previous cesarean to 63% in women with ≥3 previous cesareans and placenta previa [13]. Repeated cesarean delivery therefore contributes indirectly to future hemorrhagic risk through abnormal placentation and increasingly complex surgery.

Access to antenatal care is also uneven. A cross-country study including 2018 Romanian women identified high cesarean use, relatively limited antenatal/postnatal contact, and financial and accessibility barriers to maternity care [14]. Recent Romanian data further associated inadequate prenatal surveillance with substantially more antepartum anemia [15], while the 2025 OECD review documented persistent rural–urban inequalities, with more than 90% of hospitals and specialist outpatient facilities concentrated in urban areas [16]. These factors may increase the complexity of patients presenting to tertiary referral centers and reduce opportunities for preoperative risk optimization.

Despite extensive evidence regarding clinical predictors of transfusion, the independent association between out-of-hours cesarean delivery and perioperative transfusion remains insufficiently characterized, particularly in high-cesarean, resource-constrained settings. Therefore, the primary objective of this study was to determine whether out-of-hours cesarean delivery was independently associated with increased odds of perioperative need for red blood cell transfusion. Secondary objectives were to identify maternal, obstetric, and hematological transfusion predictors and to evaluate perioperative hemoglobin deterioration as a potential exploratory component of the observed out-of-hours association. Because out-of-hours surgery differs from routine daytime surgery in case mix as well as in staffing, the study was designed to describe an association adjusted for measured clinical factors, and not to establish a causal effect of the working period.

## 2. Materials and Methods

### 2.1. Study Design and Objectives

This retrospective, observational, single-center, unmatched case–control study included cesarean deliveries performed at a tertiary maternity hospital in Romania from 1 January 2023 to 31 December 2025. The institution records approximately 3000 deliveries annually, approximately 75% by cesarean section. This rate is well above international reference values; it reflects the national context of a cesarean rate above 50% and the institution’s role as a tertiary referral maternity unit with a large population of women with previous cesarean delivery, and it is discussed further in Section 4.5. All data were retrospectively obtained from the routinely maintained institutional clinical and laboratory database; no study-specific interventions or investigations were performed.

Primary objective. The primary aim was to determine whether out-of-hours cesarean delivery was independently associated with higher odds of perioperative red blood cell (RBC) transfusion compared with in-hours surgery. The prespecified hypothesis was that this association would persist after adjustment for relevant maternal, obstetric, and preoperative hematological confounders. Secondary objectives. The secondary aims were to identify other maternal, obstetric, hematological, and organizational predictors of transfusion; evaluate transfusion according to previous cesarean burden (0, 1, 2, or ≥3), placenta previa, and suspected placenta accreta spectrum (PAS); compare preoperative hemoglobin, postoperative/pre-transfusion hemoglobin, and ΔHb between cases and controls; examine attenuation of the out-of-hours association after addition of postoperative/pre-transfusion hemoglobin; compare transfusion timing and RBC-unit burden; assess peripartum hysterectomy and surgical reintervention for hemorrhage; and describe immediate neonatal status using the 1-min Apgar score, including Apgar < 7.

Reporting followed the STROBE recommendations for case–control studies [17]. The completed checklist is provided in Supplementary File S1 and the protocol/statistical analysis plan is in Supplementary File S2.

### 2.2. Population, Case–Control Sampling, and Exposure

The source population comprised all women undergoing cesarean delivery during the study period. Eligibility criteria were applied identically before case–control sampling. Inclusion required age ≥ 18 years, gestational age ≥ 24 weeks, cesarean delivery at the study institution, preoperative hemoglobin ≥ 10 g/dL measured before any blood product, available postoperative or intraoperative pre-transfusion hemoglobin, preoperative PT/INR/aPTT, perioperative transfusion status, and in-hours/out-of-hours classification. Exclusion criteria were preoperative hemoglobin < 10 g/dL; abnormal PT, INR, or aPTT according to institutional reference ranges or a known clinically relevant coagulopathy; cesarean delivery elsewhere followed by postpartum transfer; missing essential hemoglobin, transfusion, or exposure data; or records insufficient to determine eligibility, exposure, or outcome. The hematological restrictions were prespecified to establish a relatively preserved baseline and reduce transfusion attributable primarily to pre-existing anemia or coagulopathy.

Cases were all eligible women receiving ≥1 unit of packed RBCs intraoperatively or within 24 h after cesarean delivery. Controls were eligible non-transfused women from the same source population. Controls were selected at a 1:2 case-to-control ratio by computer-generated random sampling without replacement, stratified by calendar year (2023–2025), using a reproducible seed. No matching was performed for out-of-hours status or clinical characteristics; these were allowed to differ and were evaluated analytically. Randomization therefore was applied only to the control selection, not exposure assignment. The study size was determined by the number of eligible cases occurring during the fixed 3-year study period; all eligible cases were included, and a 1:2 control ratio was chosen because it improves statistical efficiency relative to 1:1 sampling while limiting data abstraction. No a priori power calculation was performed.

The primary exposure was timing of cesarean delivery, classified from the institutional operative register. In-hours procedures were defined as cesarean deliveries with an operative start during the regular weekday schedule, Monday through Friday between 08:00 and 15:00, excluding official public holidays. Operative start time was recorded in the operative register for all included procedures. All procedures performed outside this interval, including weekday evening/night duty periods, weekends, and public holidays, were classified as out-of-hours. The exposure classification was derived directly from the operative register and was applied independently of transfusion status. Elective/emergency classification was not included as an analytical variable because this administrative coding was not considered sufficiently reliable for clinical discrimination in the study institution. In routine practice, the “emergency” designation was frequently used for administrative or scheduling purposes, including procedures without a consistently defined degree of urgency. Its inclusion was therefore considered likely to introduce substantial exposure misclassification rather than provide a valid measure of surgical urgency.

### 2.3. Variables, Outcomes, and Data Extraction

Prespecified variables are summarized in Table 1. Previous cesarean deliveries were recorded as an exact count and categorized as 0 (unscarred uterus), 1 (single-scarred), 2 (double-scarred), or ≥3 (multiple-scarred); the exact count was retained for trend analyses. Suspected placenta accreta spectrum (PAS) was defined as a prenatal clinical diagnosis documented before cesarean delivery. The diagnosis was based either on ultrasound assessment performed at the study institution at the time of admission or on a previously established prenatal diagnosis documented by the physician responsible for antenatal follow-up and confirmed in the admission record. Histopathological confirmation was not required for classification because the variable represented the preoperative clinical suspicion available to the treating team. Preoperative hemoglobin was defined as the most recent hemoglobin concentration obtained before cesarean delivery and before administration of any blood product. Its timing relative to the onset of labor was not recorded, and labor status (spontaneous or induced onset of labor and duration of labor) was not available in the institutional database. Postoperative hemoglobin assessment was part of routine postoperative care following cesarean delivery at the study institution. For women who were not transfused intraoperatively, the first postoperative hemoglobin measurement was used. For women transfused intraoperatively, the last hemoglobin value available before administration of the first RBC unit was used, including point-of-care blood-gas hemoglobin when applicable. ΔHb was calculated as preoperative hemoglobin minus postoperative/pre-transfusion hemoglobin and was considered a complementary marker of perioperative hematological change rather than an isolated transfusion threshold.

RBC transfusion was administered according to institutional obstetric and anesthetic practice. When transfusion was considered clinically indicated, the usual initial prescription consisted of two units of packed RBC concentrate, with further blood-product administration determined according to the patient’s subsequent clinical and hematological course. Transfusion was not defined solely by a single hemoglobin threshold; the decision remained a clinical one made by the responsible obstetric and anesthetic teams. No fixed numeric hemoglobin trigger was prescribed by institutional protocol during the study period; the decision integrated hemoglobin concentration, ongoing or anticipated bleeding, hemodynamic status, and symptoms. Only packed RBC transfusion was analyzed; plasma, platelet, and fibrinogen-containing products were not included in the outcome definition.

Two investigators independently extracted, coded, compiled, and verified data using a predefined form. Discrepancies in eligibility, exposure, laboratory values, transfusion status, obstetric variables, or neonatal data were resolved by source-record review and consensus. Each record received a unique study code and direct identifiers were removed before analysis.

### 2.4. Data Management, Missing Data, and Bias Control

Data organization, coding, cleaning, and verification were performed in Microsoft Excel 2016 (Microsoft Corporation, Redmond, WA, USA) [18]; statistical analyses were performed in IBM SPSS Statistics for Windows, Version 26.0 (IBM Corp., Armonk, NY, USA) [19]. A prespecified codebook defined variable names, units, coding, permitted values, and missing-data conventions; the final analytical dataset was locked before the prespecified analyses.

Records missing data required to determine eligibility, case/control status, baseline hemoglobin/coagulation status, or the primary exposure were excluded before sampling or analysis. Missingness in nonessential covariates was reported; complete-case analysis was prespecified for ≤5% missingness, whereas >5% missingness compatible with a missing-at-random mechanism prompted multiple imputation by a fully conditional specification, with complete-case sensitivity analysis. Single-value mean/median imputation was not used. Selection bias was limited by inclusion of all eligible cases and year-stratified random control sampling from the same source population; information bias by prespecified definitions and duplicate independent extraction; exposure misclassification by avoiding poorly standardized elective/emergency coding; and confounding by prespecified multivariable and sensitivity analyses.

### 2.5. Statistical Analysis

All tests were two-sided with *p* < 0.05 for the primary analysis and 95% confidence intervals (CIs). Because the case–control ratio was fixed by design, transfusion incidence, absolute risk, and relative risk were not estimated from the analytical sample; associations were expressed as odds ratios (ORs) and adjusted ORs (aORs). The distribution of continuous variables was examined using histograms, Q-Q plots, skewness, and Shapiro–Wilk testing. To avoid reliance on distributional assumptions and to keep reporting uniform, all continuous variables were summarized as the median [interquartile range, IQR] and compared using the Mann–Whitney U test. Categorical variables were summarized as n (%) and compared using Fisher’s exact test, with the Freeman–Halton extension for tables larger than 2 × 2. Crude associations were estimated by univariable binary logistic regression.

The primary model was multivariable binary logistic regression with transfusion (case/control) as the dependent variable and out-of-hours surgery as the principal exposure, adjusted for calendar year, maternal age, BMI, parity, gestational age, multiple pregnancy, previous cesarean category, placenta previa, suspected PAS, preeclampsia, preoperative hemoglobin, platelet count, and birth weight. Age and BMI were reported per 5-unit increase, platelets per 50 × 10^9^/L, birth weight per 500 g, and preoperative hemoglobin per 1 g/dL; uterine-scar categories used an unscarred uterus as the reference. Covariates were prespecified by clinical plausibility rather than univariable significance alone. Collinearity was assessed by correlations and variance inflation factors (VIFs). A year-stratified Mantel–Haenszel pooled OR and test of heterogeneity were used as a design-consistency check. To allow assessment of overfitting, the number of events (transfused cases) and the number of estimated parameters are reported for every multivariable model; an events-per-parameter ratio of at least 10 was considered adequate.

Placenta previa and suspected PAS were retained as separate covariates because they represent clinically distinct, although related, determinants of obstetric hemorrhage. Placenta previa may increase hemorrhagic and transfusion risk independently of PAS, whereas suspected PAS identifies a subgroup with substantially greater anticipated placental invasion and operative bleeding risk. Because suspected PAS was uncommon, additional sensitivity analyses assessed the stability of the primary out-of-hours estimate after exclusion of suspected PAS and after exclusion of both suspected PAS and placenta previa.

Hematological analyses compared preoperative hemoglobin, postoperative/pre-transfusion hemoglobin, and ΔHb between cases and controls; postoperative/pre-transfusion hemoglobin was also categorized as <7.0, 7.00–7.99, 8.00–8.99, 9.00–9.99, and ≥10.0 g/dL. Gestational age and birth weight were retained as continuous covariates, and unadjusted associations were considered independent only when persisting after multivariable adjustment. An exploratory post-exposure logistic model additionally included postoperative/pre-transfusion hemoglobin to examine how adjustment for perioperative hematological status altered the estimated association between out-of-hours surgery and transfusion. Because postoperative/pre-transfusion hemoglobin was measured after the exposure and was closely related to the clinical transfusion decision, this analysis was not interpreted as a causal mediation model.

Among transfused cases, in-hours and out-of-hours groups were compared for postoperative/pre-transfusion hemoglobin, ΔHb, transfusion timing, and RBC-unit number; units were summarized as the median [IQR] and compared by the Mann–Whitney U test, with negative binomial regression prespecified exploratorily if overdispersion was present. Rare hysterectomy and reintervention outcomes used Fisher exact testing; 1-min Apgar was summarized as the median [IQR] and Apgar < 7 as the binary secondary outcome. Previous cesarean burden was examined using category-specific ORs and a trend test. Sensitivity analyses were chosen to address specific threats to the primary estimate rather than performed routinely. Excluding (1) suspected PAS and (2) suspected PAS or placenta previa tested whether the out-of-hours association was driven by the small group of women with the highest anticipated hemorrhagic risk, for whom surgery is more often planned and whose timing may therefore differ systematically from that of other cesarean deliveries; these exclusions also removed the covariates with the sparsest cells. Excluding (3) intraoperative transfusions tested whether the association depended on the subgroup in which pre-transfusion hemoglobin had a different definition (last intraoperative value rather than first postoperative value) and in which transfusion reflected acute intraoperative hemorrhage rather than a postoperative decision; this analysis was not intended to imply that intraoperative transfusions are less relevant. Because suspected PAS was uncommon among controls, the primary model was additionally refitted post hoc using Firth penalized logistic regression with profile penalized-likelihood confidence intervals in order to assess sparse-data bias.

### 2.6. Ethics, Data Protection, and Supplementary Material

The protocol was approved by the Ethics Committee of the University of Medicine and Pharmacy “Victor Babeș” Timișoara (Approval No. 132583/2026) and conducted in accordance with the World Medical Association Declaration of Helsinki, including the 2024 revision [20]. Because the study used retrospectively collected routine-care data without study-specific intervention, informed consent was waived in accordance with the ethics committee decision. Processing complied with Regulation (EU) 2016/679 (GDPR) and applicable national/institutional requirements [21]. Only prespecified data were extracted; direct identifiers were excluded, any linkage key was stored separately with restricted access, and data were held on password-protected institutional systems and reported only in aggregate. Supplementary File S1 contains the STROBE case–control checklist and Supplementary File S2 contains the study protocol and prespecified statistical analysis plan; participant selection and exclusions are shown in the flow diagram in the Results section.

## 3. Results

### 3.1. Study Population

During the study period, 9007 deliveries were recorded, of which 6773 (75.2%) were cesarean deliveries. A total of 608 women (9.0% of all cesarean deliveries) received perioperative red blood cell transfusion. After application of the predefined eligibility criteria, 401 transfused patients were retained as cases. Among the 5801 eligible non-transfused women, 802 controls were randomly selected at a 1:2 case-to-control ratio using year-stratified random sampling. The final analytical population therefore comprised 1203 women: 401 cases and 802 controls. The yearly distribution was 144 cases and 288 controls in 2023, 134 and 268 in 2024, and 123 and 246 in 2025, respectively.

**Figure 1:**
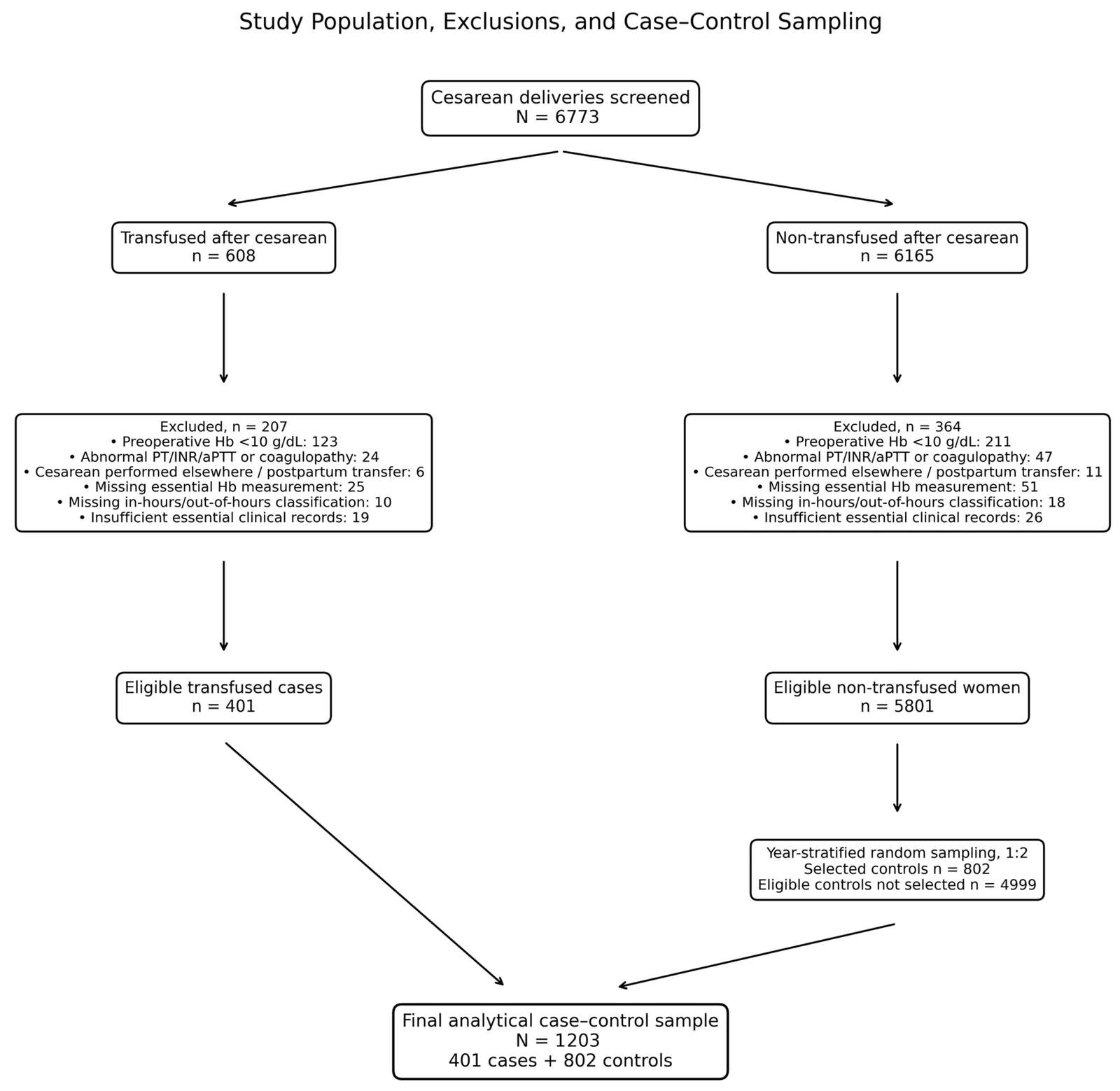
Study population, exclusions, and case–control sampling.

Patine selection process is better visualised in Figure 1.

Missing data were limited to BMI in 56 patients (4.7%), platelet count in 18 (1.5%), 1-min Apgar score in 20 (1.7%), birth weight in 11 (0.9%), and parity in 4 (0.3%). Consequently, the primary complete-case multivariable model included 1115 women (92.7% of the analytical sample), including 371 cases and 744 controls. Because missingness did not exceed the prespecified 5% threshold for any covariate, multiple imputation was not required, and all multivariable models were fitted as complete-case analyses.

### 3.2. Characteristics of Cases and Controls

Out-of-hours cesarean delivery was more frequent among transfused cases than among non-transfused controls (64.1% vs. 52.5%, *p* < 0.001). Because controls were randomly sampled from all eligible non-transfused women, the control proportion provides an unbiased estimate of the out-of-hours share of that source population (52.5%, 95% CI 49.0–56.0%). Transfused cases had a modestly lower gestational age (*p* = 0.017); birth weight was also slightly lower, achieving a difference in borderline significance on the Mann–Whitney test (*p* = 0.056) that reached significance in univariable logistic regression (*p* = 0.040). Neither variable remained independently associated with transfusion after multivariable adjustment. Multiple pregnancy, placenta previa, suspected placenta accreta spectrum (PAS), and a greater burden of previous cesarean deliveries were more frequent among cases. Among the randomly selected controls, 607/802 (75.7%) had at least one previous cesarean delivery, indicating that repeat cesarean delivery accounts for most of the institutional cesarean volume. Preoperative PT, INR, aPTT, and platelet count did not differ significantly between groups (Table 2).

### 3.3. Primary Analysis: Association Between Out-of-Hours Surgery and Transfusion

In the unadjusted analysis, out-of-hours cesarean delivery was associated with higher odds of perioperative red blood cell transfusion (OR 1.62, 95% CI 1.26–2.07; *p* < 0.001). The year-stratified Mantel–Haenszel estimate was nearly identical (pooled OR 1.62, 95% CI 1.26–2.07; *p* < 0.001), with no evidence of heterogeneity across the three study years (*p* = 0.861).

In the multivariable logistic regression model, the out-of-hours surgery remained independently associated with transfusion (aOR 2.03, 95% CI 1.45–2.84; *p* < 0.001). Placenta previa (aOR 11.40, 95% CI 4.77–27.27; *p* < 0.001), suspected PAS (aOR 93.49, 95% CI 16.47–530.75; *p* < 0.001), and lower preoperative hemoglobin were independently associated with transfusion. Each 1 g/dL increase in preoperative hemoglobin was associated with lower odds of transfusion (aOR 0.11, 95% CI 0.09–0.15; *p* < 0.001). No relevant multicollinearity was identified; the maximum variance inflation factor was 3.24. The model included 371 events and 17 estimated parameters (21.8 events per parameter) and was therefore not overparameterized. Previous cesarean burden was associated with transfusion in univariable analysis, but no scar category remained significant after adjustment; for three or more previous cesareans, the point estimate was 2.63 with a wide confidence interval, including 1 (95% CI 0.91–7.62; *p* = 0.075), reflecting the small size of this category, and it should not be interpreted as evidence of an independent effect.

Data better visualised in Table 3.

**Table 3:** Univariable and multivariable logistic regression for perioperative red blood cell transfusion.

Variable	Crude OR (95% CI)	*p*-Value	Adjusted OR (95% CI)	*p*-Value
Out-of-hours surgery	1.62 (1.26–2.07)	<0.001	2.03 (1.45–2.84)	<0.001
Maternal age, per 5 years	1.12 (0.98–1.29)	0.089	1.20 (1.00–1.44)	0.056
BMI, per 5 kg/m^2^	0.93 (0.82–1.06)	0.281	0.93 (0.79–1.09)	0.347
Parity, per 1	1.11 (1.00–1.23)	0.042	0.90 (0.70–1.16)	0.424
Gestational age, per week	0.86 (0.79–0.95)	0.003	0.93 (0.77–1.14)	0.510
Multiple pregnancy	1.85 (1.14–3.01)	0.013	1.30 (0.63–2.69)	0.479
Previous CS: 1 vs. 0	1.37 (0.99–1.89)	0.061	1.25 (0.77–2.03)	0.358
Previous CS: 2 vs. 0	1.62 (1.13–2.31)	0.009	1.46 (0.74–2.86)	0.273
Previous CS: ≥3 vs. 0	1.85 (1.15–2.96)	0.011	2.63 (0.91–7.62)	0.075
Placenta previa	11.67 (6.04–22.55)	<0.001	11.40 (4.77–27.27)	<0.001
Suspected PAS	19.99 (6.04–66.18)	<0.001	93.49 (16.47–530.75)	<0.001
Preeclampsia	1.27 (0.82–1.95)	0.288	1.31 (0.72–2.40)	0.379
Preoperative Hb, per 1 g/dL	0.17 (0.14–0.22)	<0.001	0.11 (0.09–0.15)	<0.001
Platelets, per 50 × 10^9^/L	0.95 (0.85–1.07)	0.393	0.96 (0.83–1.12)	0.639
Birth weight, per 500 g	0.89 (0.79–0.99)	0.040	1.05 (0.81–1.35)	0.731

The multivariable model was additionally adjusted for calendar year. Univariable models used all available observations: 401 events among 1203 women for every covariate except BMI (381 events/1147), parity (399/1199), platelets (396/1185), and birth weight (397/1192). The multivariable model provided a complete-case analysis (n = 1115; 371 events; 17 estimated parameters; 21.8 events per parameter). OR, odds ratio; aOR, adjusted odds ratio; CI, confidence interval; CS, cesarean section; PAS, placenta accreta spectrum.

**Figure 2:**
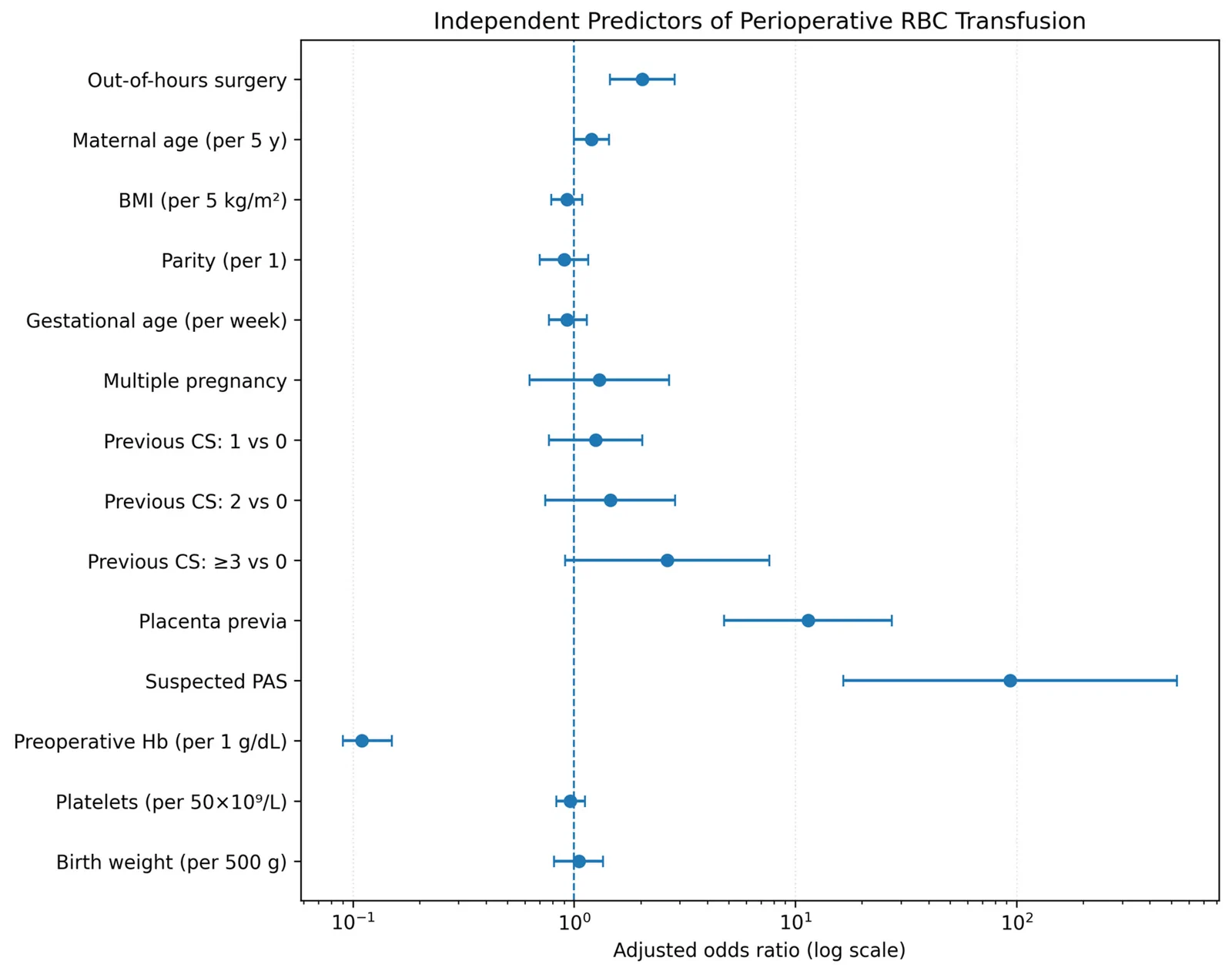
Forest plot of adjusted odds ratios for perioperative red blood cell transfusion. The horizontal axis is logarithmic. PAS, placenta accreta spectrum; CS, cesarean section.

Predictors ase visualised in Figure 2.

### 3.4. Perioperative Hemoglobin Dynamics

Transfused women had substantially lower postoperative/pre-transfusion hemoglobin concentrations than controls (median 8.60 [IQR 8.17–8.85] vs. 10.36 [9.71–11.06] g/dL; *p* < 0.001) and a greater decrease in perioperative hemoglobin (2.10 [1.67–2.58] vs. 1.21 [0.79–1.65] g/dL; *p* < 0.001). A postoperative/pre-transfusion hemoglobin concentration < 9 g/dL was recorded in 376/401 cases (93.8%), compared with 34/802 controls (4.2%; *p* < 0.001).

Among transfused patients, 18 (4.5%) had Hb < 7 g/dL, 56 (14.0%) had Hb 7.00–7.99 g/dL, and 302 (75.3%) had Hb 8.00–8.99 g/dL. Only 25 transfused patients (6.2%) had a recorded pre-transfusion hemoglobin ≥ 9 g/dL.

Data presented in Table 4.

**Table 4:** Hematological and secondary outcomes in cases and controls.

Outcome	Transfused Cases	Controls	All (n = 1203)	*p*-Value
Postoperative/pre-transfusion Hb, g/dL	8.60 [8.17–8.85]	10.36 [9.71–11.06]	9.75 [8.79–10.69]	<0.001
ΔHb, g/dL	2.10 [1.67–2.58]	1.21 [0.79–1.65]	1.51 [1.01–1.99]	<0.001
Hb < 9 g/dL	376/401 (93.8%)	34/802 (4.2%)	410/1203 (34.1%)	<0.001
Hb < 7 g/dL	18 (4.5%)	0	18 (1.5%)	
Hb 7.00–7.99 g/dL	56 (14.0%)	1 (0.1%)	57 (4.7%)	
Hb 8.00–8.99 g/dL	302 (75.3%)	33 (4.1%)	335 (27.8%)	
Hb 9.00–9.99 g/dL	17 (4.2%)	259 (32.3%)	276 (22.9%)	
Hb ≥ 10 g/dL	8 (2.0%)	509 (63.5%)	517 (43.0%)	
Peripartum hysterectomy	8 (2.0%)	0	8 (0.7%)	<0.001
Reintervention for hemorrhage	4 (1.0%)	2 (0.2%)	6 (0.5%)	0.100
Apgar at 1 min	9 [8–9]	9 [8–9]	9 [8–9]	0.686
Apgar < 7 at 1 min	22/393 (5.6%)	38/790 (4.8%)	60/1183 (5.1%)	0.575

Continuous variables are presented as the median [IQR] and compared using the Mann–Whitney U test; categorical variables are presented as n (%) and compared using Fisher’s exact test. The “All” column describes the analytical case–control sample. The overall distribution of postoperative/pre-transfusion hemoglobin categories differed significantly between cases and controls (Fisher–Freeman–Halton exact test, *p* < 0.001). Hb, hemoglobin.

### 3.5. Exploratory Post-Exposure Model Including Postoperative/Pre-Transfusion Hemoglobin

In the exploratory post-exposure model, inclusion of postoperative/pre-transfusion hemoglobin markedly attenuated the estimated association between out-of-hours surgery and transfusion, from an aOR of 2.03 in the primary model to 1.02 (95% CI 0.63–1.65; *p* = 0.922). Postoperative/pre-transfusion hemoglobin itself was strongly associated with transfusion (aOR 0.026 per 1 g/dL increase, 95% CI 0.015–0.045; *p* < 0.001). Because hemoglobin was measured after the exposure and was closely related to the clinical decision to transfuse, this analysis was considered exploratory rather than evidence of formal mediation. This model included 371 events and 18 estimated parameters (20.6 events per parameter).

### 3.6. Transfusion Characteristics According to Working Period

Of the 401 transfused women, 257 (64.1%) underwent an operation out-of-hours and 144 (35.9%) during regular working hours; these figures describe how cases were distributed between working periods and are not transfusion rates. The median number of packed red blood cell units transfused was 2 [2–2] units in both groups (*p* = 0.939). Intraoperative transfusion was numerically more frequent after in-hours than after out-of-hours surgery (20/144, 13.9% vs. 24/257, 9.3%), but the difference was not statistically significant (*p* = 0.184); in both groups, most transfusions were given after surgery.

Among transfused women, postoperative/pre-transfusion hemoglobin did not differ between those operated on out-of-hours and those operated on in-hours (median 8.60 [8.21–8.82] vs. 8.61 [8.11–8.90] g/dL; *p* = 0.405), whereas the perioperative decrease in hemoglobin was slightly larger after out-of-hours surgery (ΔHb 2.14 [1.71–2.62] vs. 1.99 [1.56–2.48] g/dL; *p* = 0.045). A pre-transfusion hemoglobin below 9 g/dL was recorded in 245/257 (95.3%) out-of-hours and 131/144 (91.0%) in-hours cases (*p* = 0.089).

Peripartum hysterectomy occurred in 5/257 (1.9%) out-of-hours cases and 3/144 (2.1%) in-hours cases (*p* = 1.000). Reintervention for hemorrhage occurred in 1/257 (0.4%) and 3/144 (2.1%), respectively (*p* = 0.134). The median 1-min Apgar score was 9 [8–9] in both groups (*p* = 0.566). Among transfused cases with available Apgar data, an Apgar score < 7 occurred in 19/251 (7.6%) out-of-hours deliveries compared with 3/142 (2.1%) in-hours deliveries (*p* = 0.023).

### 3.7. Sensitivity Analyses

The association between out-of-hours surgery and transfusion remained stable in all prespecified sensitivity analyses (Table 5). In the post hoc Firth-penalized model, the out-of-hours estimate was essentially unchanged (aOR 2.00, 95% CI 1.44–2.79; *p* < 0.001). The corresponding penalized estimates were attenuated but remained strong for placenta previa (aOR 10.43, 95% CI 4.61–25.50) and for suspected PAS (aOR 78.67, 95% CI 15.85–466.45).

**Figure 3:**
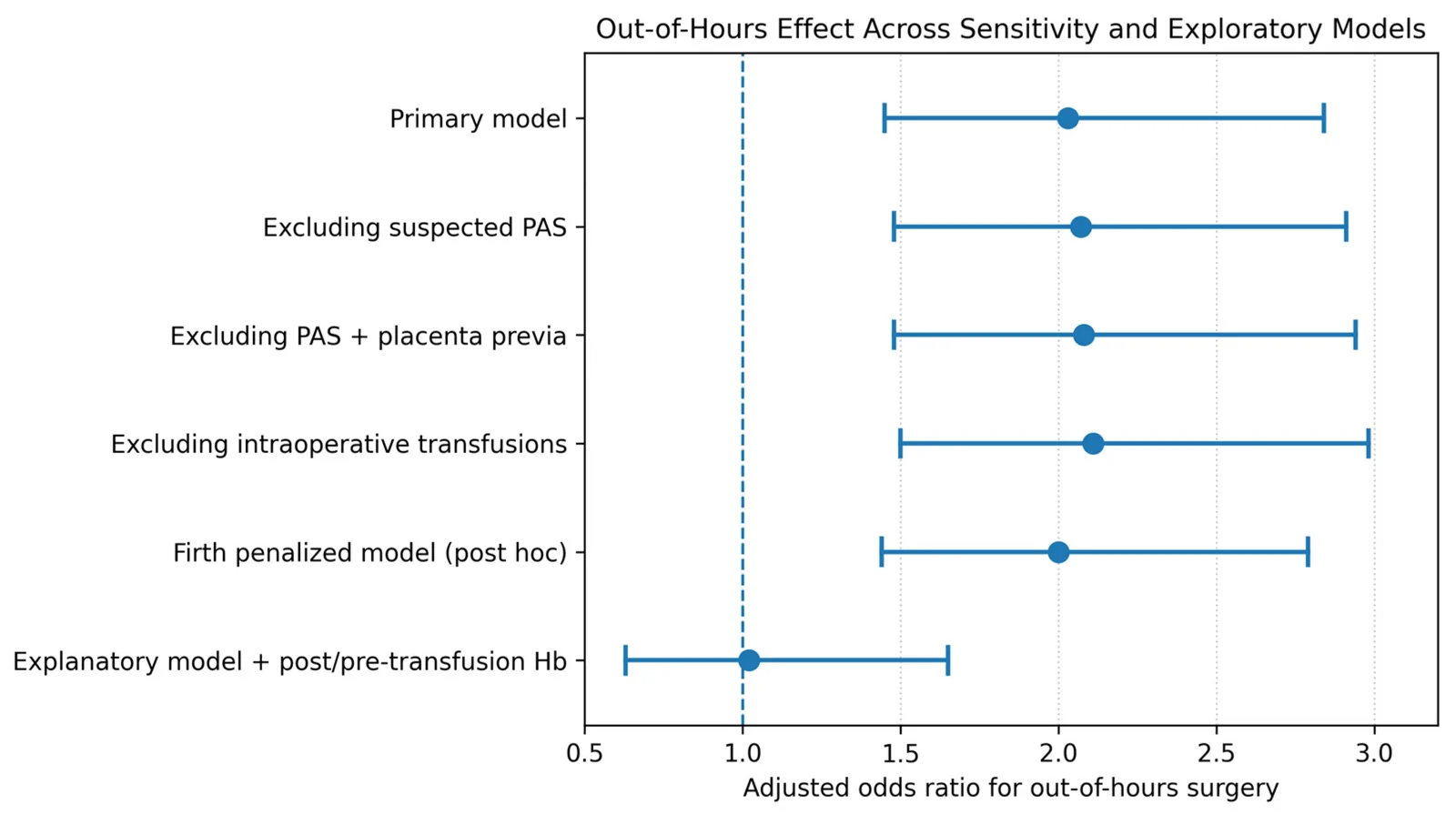
Adjusted association between out-of-hours cesarean delivery and transfusion across the primary, sensitivity, exploratory, and post hoc Firth-penalized models. PAS, placenta accreta spectrum; Hb, hemoglobin.

The adjusted association between out-of-hours surgery and transfusion therefore remained consistent after removal of major placental hemorrhagic conditions and intraoperative transfusion cases, whereas inclusion of postoperative/pre-transfusion hemoglobin substantially altered the effect estimate (Figure 3).

## 4. Discussion

### 4.1. Principal Findings

The principal finding of this study was that cesarean delivery performed out-of-hours was associated with approximately two-fold higher odds of perioperative red blood cell transfusion after adjustment for the measured maternal, obstetric, placental, and hematological factors (aOR 2.03, 95% CI 1.45–2.84). This association remained essentially unchanged when women with suspected placenta accreta spectrum (PAS) or placenta previa were excluded, and when the analysis was restricted to women transfused after surgery, that is, after excluding the 11% of cases transfused intraoperatively. This association should, however, be read as a difference between two clinically different groups of women rather than as an effect of clock time: out-of-hours cesarean deliveries are largely unplanned, and cesarean indication, urgency, and labor characteristics were not available for adjustment. However, after postoperative/pre-transfusion hemoglobin was introduced into the exploratory post-exposure model, the association was almost completely attenuated (aOR 1.02, 95% CI 0.63–1.65). Because postoperative/pre-transfusion hemoglobin is closely linked to the clinical decision to transfuse, substantial attenuation after its inclusion was expected and should not be over-interpreted. The pattern is compatible with greater perioperative hematological deterioration after out-of-hours surgery, but it does not establish this mechanism, and mediation cannot be inferred from the present retrospective design.

Two further findings among transfused women help to interpret this association. First, pre-transfusion hemoglobin was similar in women operated on out-of-hours and in-hours (median 8.60 vs. 8.61 g/dL), which argues against a more liberal transfusion threshold during duty periods as the sole explanation. Second, the perioperative decrease in hemoglobin was slightly larger after out-of-hours surgery (median 2.14 vs. 1.99 g/dL; *p* = 0.045). This difference is small, unadjusted, of borderline significance, and one of several secondary comparisons; it is compatible with somewhat greater blood loss after out-of-hours surgery, but the present data cannot distinguish greater blood loss from differences in case mix or unmeasured acuity. Future studies should therefore prioritize objective blood-loss measures and perioperative processes rather than transfusion alone.

Among transfused women, a 1-min Apgar score < 7 was more frequent after out-of-hours delivery (7.6% vs. 2.1%; *p* = 0.023). This comparison was unadjusted, based on a few events, and is one of several secondary comparisons; it may reflect greater fetal compromise among urgent out-of-hours deliveries rather than the effect of the working period itself, and it should be regarded as hypothesis-generating.

The magnitude of the observed out-of-hours association is somewhat greater than, but directionally consistent with, several large population studies. Nam et al., analyzing 90,072 deliveries, reported higher risks of severe maternal morbidity (SMM) for weekday nighttime (RR 1.26) and weekend/holiday deliveries (RR 1.58); importantly, blood transfusion itself was increased both at night (RR 1.32, 95% CI 1.12–1.56) and during weekends/holidays (RR 1.60, 95% CI 1.27–2.03) [4]. A subsequent Korean nationwide analysis of more than 3 million deliveries similarly found the greatest SMM risk during nighttime delivery (aRR approximately 1.40), with stronger effects in smaller-city hospitals and hospitals with 100–499 beds, where weekend-night SMM reached an aRR of 1.83 [5]. In a U.S. population of more than 3 million otherwise low-risk births, Kim and Selya found a smaller but consistent weekend effect (OR 1.13, 95% CI 1.11–1.14) across maternal and neonatal outcomes that included transfusion [6].

Not all studies demonstrate an adverse out-of-hours effect. Yee et al. evaluated 7917 women with postpartum hemorrhage across 25 U.S. hospitals and found similar adjusted hemorrhage-related maternal morbidity after nighttime and daytime delivery (15.5% vs. 17.5%; aOR 0.89, 95% CI 0.77–1.03) [22]. Similarly, a 2026 multicenter Chinese study of 9097 cesarean deliveries found higher rates of preterm delivery, low birth weight, and NICU admission on weekends but no significant increase in postpartum hemorrhage; the authors specifically cautioned that weekend findings may reflect case mix and urgent indications rather than differences in quality of care [23]. The heterogeneous literature therefore supports interpreting out-of-hours status as a marker of a complex interaction between patient acuity and hospital organization rather than as an intrinsically hazardous period.

### 4.2. Out-of-Hours Care, Staffing, and 24-Hour Duty Shifts

This distinction is particularly relevant to our institution, where obstetric duty periods extend continuously for 24 h and out-of-hours activity is generally covered by fewer personnel than routine daytime activity. Our study did not measure hours already worked by the surgical team, individual staffing levels, or fatigue; therefore, these factors cannot be considered demonstrated mechanisms. Nevertheless, existing evidence provides a plausible organizational framework.

Aiken et al. examined 24,506 unscheduled deliveries and found no overall difference between day and night shifts. However, the risk of blood loss ≥ 1.5 L and severe neonatal acidosis varied by approximately 30–40% according to the number of hours already worked, with the highest risk after approximately 9–10 h of a shift [9]. This observation is particularly relevant to a 24-h duty model because a binary day/night comparison may underestimate the effect of cumulative workload. A larger systematic review (134 studies, >1.6 million cases) confirmed that fatigue measurably impairs surgical performance [24], although a comparably large anesthesiology cohort found no significant, and only directionally consistent, harm from extended work periods [25]. A natural experiment following resident duty-hour restriction at three Toronto teaching hospitals found no change in overall obstetric outcomes but a significant increase in transfusion/postpartum hemorrhage (OR 1.23, 95% CI 1.07–1.41) and surgical/obstetric complications (OR 1.19), indicating that shorter individual shifts do not translate directly into safer perioperative care [26].

Conversely, Tavares et al. studied 9143 deliveries in a tertiary center specifically characterized by 24/7 presence of experienced medical staff and found no clinically relevant day–night differences in maternal or neonatal outcomes, including obstetric emergencies [8]. These findings provide an important counterpoint to our results: they suggest that the adverse signal associated with out-of-hours delivery is potentially modifiable and may depend more on differences in staffing and senior expertise than on clock time itself.

Large health-system analyses support this interpretation. The 2023 Korean nationwide study found that the association between nighttime delivery and SMM was stronger in hospitals with fewer resources and in smaller cities [5]. In another analysis of more than two million Texas deliveries, maternal complications increased during night shifts (OR 1.21), weekends (OR 1.09), and holidays (OR 1.29), periods characterized by reduced clinical staffing and different staff composition [27]. Our adjusted OR of approximately 2.0 therefore does not show that operating at night causes hemorrhage. It is more plausibly explained by a combination of higher clinical acuity in unplanned out-of-hours surgery and differences in organizational capacity between regular-hours and duty-period care, which the present design cannot separate.

Several mechanisms could plausibly explain the higher adjusted odds of transfusion after out-of-hours surgery in our setting, and they are not mutually exclusive. The first is case mix and acuity. Out-of-hours cesarean deliveries are mostly unplanned and more often follow labor, prolonged or failed induction, intrapartum complications, or acute antepartum events, all of which increase the risk of uterine atony and hemorrhage regardless of who performs the operation. For example, a woman admitted for induction of labor in the morning may undergo cesarean delivery at night after prolonged labor; the night-time surgery, the subsequent atony, and the transfusion would then share a common cause. Chorioamnionitis associated with prolonged labor could act in the same way. The second is organizational: smaller duty teams, less immediate availability of senior obstetric and anesthetic staff, longer time already spent on duty, and slower access to laboratory and blood-bank services may delay the recognition and control of bleeding. The third is postoperative surveillance, as fewer ward staff at night may lead to later detection of ongoing bleeding. Because indication, urgency, labor characteristics, and staffing were not recorded in our dataset, the relative contribution of these mechanisms cannot be determined.

### 4.3. Obstetric and Hematological Predictors of Transfusion

The clinical predictors identified in our study are strongly concordant with the previous transfusion literature. In a systematic review and meta-analysis of 22 studies, including 426,094 cesarean deliveries, Iqbal et al. found placenta previa to be among the strongest predictors of transfusion (OR 9.54, 95% CI 7.23–12.59), together with placental abruption (OR 6.77), multiple pregnancy (OR 1.60), preterm labor (OR 3.34), hypertensive disorders (OR 1.81), and unbooked pregnancy (OR 2.42) [1]. Our adjusted estimate for placenta previa (aOR 11.40) is therefore remarkably close to the pooled estimate from the literature.

Recent placenta previa cohorts provide similar evidence. In a 2026 study of women with major placenta previa, 18.2% required transfusion, and previous cesarean delivery increased transfusion odds by approximately 4.6-fold [28]. Earlier case–control data in placenta previa similarly identified previous cesarean delivery, major/anterior previa, preoperative bleeding, and anemia as independent transfusion predictors [29]. In women undergoing a third or higher-order cesarean, Spiegelman et al. reported a transfusion rate of only 1.4% after placenta previa was excluded, compared with a 68.8% probability of transfusion in patients with previa, illustrating that the placental disorder rather than scar number alone may dominate hemorrhagic risk [30].

This is consistent with our finding that increasing uterine scar burden showed an association in unadjusted analyses but became substantially weaker after adjustment for placenta previa and PAS. Klahr et al. analyzed 3582 cesareans and found progressively increasing adverse outcomes with additional procedures; however, when women without placenta previa were examined separately, major morbidity differed little across cesarean-number groups, whereas morbidity increased markedly with repeated cesareans in the presence of previa [31]. Thus, the relationship between repeat cesarean and transfusion is likely mediated substantially through abnormal placentation.

The extremely high aOR observed for suspected PAS in our model should be interpreted in light of its wide confidence interval rather than as an exact effect size. With only three exposed controls, this estimate is susceptible to sparse-data bias, which tends to inflate odds ratios away from the null; in a Firth-penalized model the estimate fell to an aOR of 78.67 (95% CI 15.85–466.45), so its magnitude should be regarded as unstable even though the association remains strong. The direction of the association is nevertheless biologically credible. A separate multicenter registry found previous cesarean to be the strongest PAS predictor in the presence of placenta previa, with aRR 5.34 after one previous cesarean and 16.5 after two or more [32].

PAS also illustrates why adequate prenatal diagnosis matters. A 2023 meta-analysis of 31 studies found that prenatal PAS diagnosis was associated with fewer emergency cesareans (OR 0.37), lower blood loss, and approximately 1.96 fewer RBC units transfused compared with cases not diagnosed before delivery [33]. A 2024 meta-analysis further showed that emergency delivery in PAS was associated with approximately 595 mL additional blood loss, 2.3 additional RBC units, and a 3.8-fold higher odds of receiving >4 RBC units compared with scheduled delivery [34]. These findings are particularly relevant to referral centers receiving incompletely investigated high-risk pregnancies.

Preoperative hemoglobin was the other major predictor of transfusion in our cohort. Despite restricting inclusion to Hb ≥ 10 g/dL, each additional 1 g/dL was strongly associated with lower transfusion odds, in close agreement with recent studies. In a cohort of 43,869 cesarean deliveries with a transfusion rate of 3.3%, Okada et al. found that women with a larger prepartum red-cell mass were substantially less likely to be transfused, whereas placental complications were associated with approximately six-fold higher odds of transfusion [3]. Closer to our setting, a 2026 Romanian study of 932 pregnant women reported a transfusion rate of 5.2% and found preoperative hemoglobin to be the strongest predictor of transfusion (OR 0.216 per 1 g/dL increase); a preoperative hemoglobin of 10.9 g/dL best separated transfused from non-transfused women (AUC 0.875) [2]. Our findings therefore reproduce, in a different analytical framework, a very recent observation from another Romanian tertiary setting. Contemporary prediction models reinforce these predictors: a secondary analysis of a multicenter U.S. tranexamic-acid trial identified preoperative hemoglobin, platelet count < 100,000, aspirin use, chorioamnionitis, oxytocin duration, and second-stage cesarean delivery as independent transfusion predictors (AUC 0.79) [35], while a Turkish weighted risk score built on placenta previa, coagulopathy, and hemoglobin/platelet thresholds showed similar discrimination (AUC 0.838) [36]. Such bedside scores could complement out-of-hours preparedness protocols by flagging high-risk patients regardless of working period.

The strong relationship between postoperative/pre-transfusion Hb and transfusion was expected. Most transfused women had a recorded pre-transfusion hemoglobin between 8.0 and 8.99 g/dL. These concentrations are higher than thresholds commonly applied to stable, non-bleeding patients under restrictive patient blood-management strategies [37]. However, transfusion decisions in obstetric hemorrhage depend on active bleeding, hemodynamic status, anticipated ongoing blood loss, symptoms, and serial hematological assessment rather than hemoglobin concentration alone, consistent with current WHO guidance on obstetric hemorrhage management [38]. Because these decision-level variables were not uniformly available in the retrospective dataset, the appropriateness or restrictiveness of individual transfusion decisions cannot be determined from the present study.

### 4.4. Gestational Age and Birth Weight

Transfused cases had a modestly lower gestational age and birth weight in unadjusted comparisons; however, neither variable remained independently associated with transfusion after multivariable adjustment. This is worth noting because higher birth weight is itself associated with an increased risk of hemorrhage: in a 2019 meta-analysis, birth weight > 4000 g was associated with approximately two-fold higher odds of PPH (OR 2.05) and birth weight > 4500 g was associated with more than three-fold higher odds (OR 3.15) [39]. The large 2025 Lancet meta-analysis of 327 studies similarly identified macrosomia > 4500 g among strongly associated PPH risk factors [40].

The lower birth weight of transfused women in our unadjusted comparison therefore runs opposite to what the macrosomia literature would predict and is best explained by the case mix. Earlier gestational age and lower birth weight in transfused cases likely reflect the high-risk case mix, including previa/PAS, hypertensive disease, multiple gestation, and earlier delivery. This interpretation is also supported by Iqbal et al., who found preterm labor to be associated with a 3.34-fold increase in transfusion odds [1]. The disappearance of both gestational age and birth weight effects after multivariable adjustment in our model supports confounding rather than an independent causal relationship.

### 4.5. Romanian Context: High Cesarean Burden and Antenatal Care Gaps

An important feature of the present setting is the approximately 75% institutional cesarean delivery rate. This figure is high but should be considered within the broader Romanian context. This rate has risen steadily, from 40.1% in 2013 to 46.9% in 2018 to 52.9% in 2020, with cesarean use disproportionately higher in wealthier regions, the capital, and private hospitals, patterns attributed in part to financial incentives under the national DRG payment system [10]. More recent international surveillance estimated the Romanian national rate at 51.4% in 2023, placing Romania sixth among the countries included in the 2026 international comparison [11]. Thus, although the present institutional rate exceeds the national mean, it occurs within a healthcare system already characterized by exceptionally high cesarean use. Two features of our institution add to this. First, as a tertiary referral maternity unit, it serves a referral area covering roughly one-fifth of Romania’s territory, with an estimated population of 2–3 million, and receives a disproportionate share of that area’s high-risk pregnancies, including women with placenta previa, suspected PAS, multiple pregnancy, and hypertensive disorders. Second, repeat procedures account for most of the cesarean volume: three-quarters of the randomly selected controls (75.7%) had at least one previous cesarean delivery, so high primary cesarean rates in earlier years are carried forward through repeat surgery. We do not regard this rate as optimal; it exceeds international benchmarks, and its implications for the generalizability of our findings are acknowledged in Section 4.7.

A high tertiary-center rate should not be compared mechanically with a universal hospital target. The WHO explicitly does not recommend a fixed cesarean rate for individual hospitals because referral centers treat disproportionately complex populations [12]. Nevertheless, repeated cesarean exposure has cumulative implications. The progressive accumulation of single-, double-, and multiple-scarred uteri increases the future population susceptible to previa, PAS, adhesions, hysterectomy, and major hemorrhage. This relationship is supported both by PACCRETA [13] and by contemporary repeat-cesarean cohorts [31]. Consequently, high cesarean utilization affects not only the index delivery but also the risk structure of subsequent pregnancies returning to the same maternity system.

The second relevant Romanian characteristic is unequal access to consistent antenatal care. In a cross-country analysis including 2018 Romanian women, Miteniece et al. identified high cesarean use, relatively few antenatal/postnatal contacts, financial barriers, staff shortages, and access problems related to distance, referral pathways, and restricted availability [14]. The 2025 OECD review similarly documented substantial geographical inequalities: 54% of the Romanian population lived in rural areas in 2023, while >90% of hospitals and independent specialist clinics were located in urban settings; unmet healthcare needs were approximately 1.5-fold higher in rural than urban populations [16].

Recent Romanian obstetric data are consistent with these structural observations. Zampieri and Ionescu reported antepartum anemia in 60.9% of women without prenatal care versus 39.3% of those receiving prenatal care, with lower postpartum hemoglobin levels among inadequately monitored women [15]. Although their small cohort did not demonstrate a statistically significant difference in transfusion, the direction is consistent with larger international evidence. In the French PreCARE cohort of 9117 pregnancies, attendance at <50% of recommended antenatal visits was associated with severe maternal morbidity (aOR 2.40) and severe perinatal morbidity (aOR 2.27) [41]. Likewise, the cesarean transfusion meta-analysis found unbooked pregnancies associated with more than twice the odds of transfusion (OR 2.42) [1].

These observations provide a relevant framework for tertiary Romanian hospitals receiving patients with incomplete surveillance. Delayed recognition of anemia, placenta previa, or PAS reduces the opportunity for antenatal correction of hemoglobin, planned delivery, blood-product preparation, and multidisciplinary surgical planning. Importantly, antenatal-care adequacy was not measured in our dataset and therefore cannot be claimed as an explanation for the observed out-of-hours effect; it should instead be considered an important contextual factor for future prospective studies.

### 4.6. Clinical and Organizational Implications

The practical implication is not that cesarean deliveries should be avoided out-of-hours; urgent obstetric surgery is inherently continuous. The appropriate target is equivalent preparedness regardless of working period.

In a resource-constrained maternity unit, feasible interventions include systematic identification of previa/PAS and scar burden, optimization of antenatal hemoglobin, predefined blood-bank notification criteria, readily available crossmatched blood for high-risk cases, structured hemorrhage protocols, and early escalation to senior obstetric and anesthetic staff. The PAS literature strongly supports planned preparation: prenatal diagnosis is associated with lower blood loss and fewer RBC units transfused [33], whereas emergency PAS surgery substantially increases hemorrhage and major transfusion requirements [34]. Blood-delivery barriers documented in other resource-constrained obstetric settings support this approach [42]; viscoelastic-guided transfusion (e.g., ROTEM/TEG) can further individualize decisions but remains limited by costs and training in low-resource contexts [43]. Prophylactic tranexamic acid in women at increased hemorrhage risk reduces blood loss and transfusion need in randomized trials, offering a low-cost adjunct particularly suited to resource-constrained settings [44]. Recent evidence syntheses reinforce structured hemorrhage-management strategies more broadly [45].

### 4.7. Strengths, Limitations, and Future Directions

Strengths include the focused case–control design, inclusion of all eligible transfusion cases, year-stratified random selection of two controls per case, restriction to women with Hb ≥ 10 g/dL and normal preoperative coagulation, standardized outcome definitions, independent data extraction by two investigators, and consistent results across multiple sensitivity analyses.

A major limitation is the retrospective single-center design. Although exact operative start time was available, out-of-hours status was analyzed as a binary variable, and finer time-of-day or day-of-week patterns were not examined. The duration of the duty period already completed, alongside staffing numbers, operator seniority, anesthetic team composition, quantitative blood loss, intraoperative hemodynamics, uterotonic requirements, and exact clinical indication for transfusion were unavailable. These unmeasured factors prevent attribution of the observed association to fatigue, understaffing, or any other individual organizational mechanism. Residual confounding remains possible. Labor status before surgery (spontaneous or induced onset and duration of labor), intrapartum infection, and the seniority of the operating obstetrician and anesthetist were also unavailable. These are plausible confounders, because prolonged or induced labor and intrapartum infection predispose to atony and are more likely to culminate in cesarean delivery outside regular hours, and duty-period teams may include fewer senior clinicians. Only packed RBC transfusion was analyzed; plasma, platelet, and fibrinogen administration and the use of viscoelastic testing were not evaluated.

Cesarean urgency represents an important potential confounder of the relationship between operative timing and transfusion. However, elective/emergency coding was not included because the institutional administrative designation did not reliably distinguish a true clinical urgency: “emergency” status was frequently assigned for organizational or scheduling reasons and was therefore considered susceptible to substantial misclassification. Consequently, residual confounding by clinical urgency and by the underlying indication for surgery cannot be excluded, and we regard this as the most important limitation of the study. Planned cesarean deliveries take place almost exclusively during regular hours, whereas out-of-hours procedures are largely unplanned; the clinical conditions that lead to unplanned surgery may themselves increase hemorrhagic risk, and part of the observed association, possibly a large part, may reflect these conditions rather than the working period. Future prospective studies should use a standardized urgency classification based on the actual clinical indication and required decision-to-delivery interval rather than routine administrative coding. In addition, the distribution of vaginal and cesarean deliveries by working period was not extracted, so we could not examine whether the cesarean rate itself differed between regular hours and duty periods. The very high institutional cesarean rate, with a large proportion of repeat procedures, also limits the generalizability of our findings to maternity units with lower cesarean rates.

The case–control design also precludes estimation of transfusion incidence directly from the analytical sample, and the working-period distribution of the full source population was not extracted, so the out-of-hours share of this population is estimated from the randomly sampled controls rather than counted directly. PAS was based on clinical suspicion rather than uniform histological confirmation, and the suspected PAS estimate rested on few exposed controls and is correspondingly imprecise. Postoperative/pre-transfusion hemoglobin had to be defined in two ways: for women transfused during surgery, it was the last value measured before the first RBC unit, and for all other women, it was the first postoperative value. More importantly, this value is the main information clinicians use when deciding whether to transfuse. Adjusting for it is therefore close to adjusting for the outcome itself, and the exploratory model would be expected to weaken almost any association between an exposure and transfusion; the attenuation seen in this model should not be read as proof that greater hemoglobin loss explains the out-of-hours association. The exclusion of women with preoperative Hb < 10 g/dL and abnormal coagulation improves baseline comparability but limits generalizability to women entering surgery with significant hematological abnormalities. Decision-level clinical variables underlying individual transfusion decisions (active bleeding, hemodynamic status, and serial clinical assessment) were not uniformly available, so the restrictiveness of local transfusion practice relative to international strategies could not be determined; neonatal assessment was also limited to the 1-min Apgar score.

A prospective study is needed and should, above all, record the cesarean indication and its clinical urgency, labor characteristics (spontaneous or induced onset and duration of labor), and intrapartum infection. It should also collect exact operative timing, hours already worked by the surgical team, number and seniority of available personnel, quantified blood loss, fluid administration, hemodynamic variables, uterotonics, time to hemorrhage control, blood-product availability, exact transfusion indication, and adequacy of antenatal surveillance. Such data would show how much of the out-of-hours association is explained by the clinical condition of the patient, how much by the volume of blood lost, and how much by staffing, fatigue, and local transfusion practice.

## 5. Conclusions

Out-of-hours cesarean delivery was associated with approximately two-fold higher odds of perioperative red blood cell transfusion after adjustment for measured maternal, obstetric, placental, and hematological factors. Because cesarean indication, urgency, and labor characteristics were not available, this association may partly reflect the different clinical profile of unplanned out-of-hours surgery rather than the working period itself. This finding is consistent with population studies showing increased transfusion and severe maternal morbidity during nights, weekends, and holidays, although other well-staffed centers have reported no day–night difference. The association was attenuated after accounting for postoperative/pre-transfusion hemoglobin; because this measure is closely linked to the transfusion decision, this finding is compatible with, but does not establish, a contribution of greater perioperative hematological deterioration.

Placenta previa, suspected PAS, and lower baseline hemoglobin were the principal clinical predictors and closely reproduced associations reported in large cohorts and meta-analyses. These findings are particularly relevant in the Romanian context of very high cesarean utilization, frequent scarred uteri, unequal access to antenatal care, and resource constraints during duty periods. Improving preoperative risk recognition and reducing differences in preparedness between routine and out-of-hours care may represent practical targets for reducing transfusion requirements. Before such measures are introduced, however, a prospective study that records cesarean indication, urgency, and the reasons for urgency is needed to establish which components of the out-of-hours association are modifiable.

## Figures and Tables

**Table 1:** Prespecified study variables, coding, and analytical role.

Domain	Variable	Scale/Coding	Analytical Role
Maternal	Age	years	Covariate
Maternal	BMI	kg/m^2^	Covariate
Obstetric	Parity	count	Covariate
Obstetric	Gestational age	weeks	Covariate
Obstetric	Multiple pregnancy	0/1	Covariate
Obstetric	Previous cesarean deliveries	exact count; 0/1/2/≥3	Covariate/trend
Obstetric	Placenta previa	0/1	Covariate
Obstetric	Suspected PAS	0/1	Covariate/sensitivity
Obstetric	Preeclampsia	0/1	Covariate
Neonatal	Birth weight	g	Covariate
Hematological	Preoperative Hb	g/dL	Eligibility/covariate
Hematological	Platelets	×10^9^/L	Covariate
Coagulation	PT, INR, aPTT	continuous	Eligibility
Hematological	Postoperative/pre-transfusion Hb	g/dL	Secondary/exploratory
Hematological	ΔHb	g/dL	Secondary
Organizational	Out-of-hours surgery	0/1	Primary exposure
Outcome	RBC transfusion within 24 h	0/1	Primary outcome
Outcome	RBC units/timing	count/category	Secondary
Outcome	Hysterectomy/reintervention	0/1	Secondary
Neonatal	Apgar at 1 min; Apgar < 7	0–10; 0/1	Secondary

The primary outcome was perioperative RBC transfusion (≥1 packed RBC unit intraoperatively or within 24 h). Secondary maternal outcomes were postoperative/pre-transfusion hemoglobin, ΔHb, transfusion timing and RBC-unit number, peripartum hysterectomy, and reintervention for hemorrhage; ICU admission was excluded because local admission practice was not a reliable severity endpoint. The neonatal outcome was the routinely documented 1-min Apgar score, analyzed descriptively and as <7 versus ≥7; it was not used as a predictor in the maternal transfusion model.

**Table 2:** Maternal, obstetric, and preoperative characteristics.

Variable	Transfused Cases (n = 401)	Controls (n = 802)	All (n = 1203)	*p*-Value
Out-of-hours surgery	257 (64.1%)	421 (52.5%)	678 (56.4%)	<0.001
Maternal age, years	31.1 [28.7–33.9]	30.7 [27.7–33.7]	30.9 [28.0–33.8]	0.066
BMI, kg/m^2^	26.8 [23.4–30.4]	27.4 [23.9–30.4]	27.1 [23.9–30.4]	0.253
Parity	1 [1–2]	1 [1–2]	1 [1–2]	0.027
Gestational age, weeks	38.1 [37.2–39.0]	38.3 [37.5–39.0]	38.2 [37.4–39.0]	0.017
Multiple pregnancy	33 (8.2%)	37 (4.6%)	70 (5.8%)	0.013
Previous cesarean deliveries				0.023
0—unscarred uterus	71 (17.7%)	195 (24.3%)	266 (22.1%)	
1—single-scarred uterus	177 (44.1%)	356 (44.4%)	533 (44.3%)	
2—double-scarred uterus	110 (27.4%)	187 (23.3%)	297 (24.7%)	
≥3—multiple-scarred uterus	43 (10.7%)	64 (8.0%)	107 (8.9%)	
Placenta previa	56 (14.0%)	11 (1.4%)	67 (5.6%)	<0.001
Suspected PAS	28 (7.0%)	3 (0.4%)	31 (2.6%)	<0.001
Preeclampsia	36 (9.0%)	58 (7.2%)	94 (7.8%)	0.306
Birth weight, g	2985 [2601–3298]	3020 [2707–3359]	3014 [2671–3351]	0.056
Preoperative Hb, g/dL	10.60 [10.27–10.99]	11.61 [11.00–12.23]	11.20 [10.62–11.94]	<0.001
Platelets, ×10^9^/L	232 [190–267]	231 [194–267]	231 [194–267]	0.633
PT, s	12.02 [11.65–12.46]	12.09 [11.68–12.55]	12.08 [11.67–12.52]	0.120
INR	0.97 [0.93–1.00]	0.96 [0.92–0.99]	0.96 [0.92–1.00]	0.105
aPTT, s	29.35 [27.57–31.16]	29.59 [27.82–31.18]	29.53 [27.73–31.17]	0.509

Continuous variables are presented as median [IQR] and compared using the Mann–Whitney U test; categorical variables are presented as n (%) and compared using Fisher’s exact test (Freeman–Halton extension for previous cesarean deliveries). The “All” column describes the analytical case–control sample; because cases were deliberately oversampled, it does not estimate the characteristics of the source cesarean population. PAS, placenta accreta spectrum.

**Table 5:** Sensitivity analyses for the association between out-of-hours surgery and transfusion.

Model	n	Events	df	EPV	Adjusted OR (95% CI)	*p*-Value
Primary multivariable model	1115	371	17	21.8	2.03 (1.45–2.84)	<0.001
Excluding suspected PAS	1085	344	16	21.5	2.07 (1.48–2.91)	<0.001
Excluding PAS and placenta previa	1044	312	15	20.8	2.08 (1.48–2.94)	<0.001
Excluding intraoperative transfusions	1075	331	17	19.5	2.11 (1.50–2.98)	<0.001
Firth penalized primary model (post hoc)	1115	371	17	21.8	2.00 (1.44–2.79)	<0.001
Exploratory model + postoperative/pre-transfusion Hb	1115	371	18	20.6	1.02 (0.63–1.65)	0.922

Events are the transfused cases contributing to each model. df is the number of regression coefficients estimated in addition to the intercept: out-of-hours surgery, calendar year (2), maternal age, BMI, parity, gestational age, multiple pregnancy, previous cesarean category (3), placenta previa, suspected PAS, preeclampsia, preoperative Hb, platelet count, and birth weight. Covariates became constant after exclusion, and the exploratory model added postoperative/pre-transfusion Hb. EPV, events per parameter (events/df); PAS, placenta accreta spectrum; OR, odds ratio; CI, confidence interval; Hb, hemoglobin.

## Data Availability

The data presented in this study are available on request from the corresponding author.

## Supplementary Materials

The following supporting information can be downloaded at https://www.mdpi.com/article/10.3390/jcm15218646/s1. Supplementary File S1: STROBE checklist for case–control studies; Supplementary File S2: Study protocol and prespecified statistical analysis plan.
