# Supplementary material for: Predictors of Blood Transfusion Following Cesarean Delivery in Resource-Constrained Settings: The Impact of Out-of-Hours Surgery
Source: J Clin Med. 2026 Nov 6;15(21):8646. doi: 10.3390/jcm15218646 (PMC13764284; doi:10.3390/jcm15218646)
Supplement: Supplementary file 1 [file jcm-15-08646-s001.zip › Supplementary_File_S1_STROBE_Case_Control_Checklist.pdf]

Supplementary File S1

STROBE Case–Control Checklist

Predictors of Blood Transfusion Following Cesarean Delivery in Resource-Constrained Settings: The Impact of Out-of-Hours Surgery

Victor Bogdan Buciu; Andrei Pescaru; Denis Mihai Șerban; Sebastian Ciurescu; Răzvan Roșca

Reporting framework: STROBE Statement: Checklist for case-control studies.

Section	Item	STROBE requirement (condensed)	Where addressed in manuscript	Status
**Title and abstract**	1a	Identify the observational case-control design in the title or abstract.	Abstract — Methods; manuscript title/abstract	Yes
**Title and abstract**	1b	Provide a balanced abstract summary of methods and principal findings.	Abstract	Yes
**Introduction**	2	Explain the scientific background and rationale.	Introduction, paragraphs 1–5	Yes
**Introduction**	3	State objectives and prespecified hypotheses.	End of Introduction; Methods 2.1	Yes
**Methods**	4	Present the key design elements early.	Methods 2.1	Yes
**Methods**	5	Describe setting, location, and relevant study/data-collection dates.	Methods 2.1–2.2	Yes
**Methods**	6a	Give eligibility criteria, sources/methods of case ascertainment and control selection, and rationale for case/control choice.	Methods 2.2; Figure 1; Supplementary File S2	Yes
**Methods**	6b	For matched studies, report matching criteria and number of controls per case.	Not applicable: unmatched design. A 1:2 sampling ratio was used without matching.	N/A
**Methods**	7	Define outcomes, exposure, predictors, confounders, effect modifiers, and diagnostic criteria where applicable.	Methods 2.3; Supplementary File S2, Table S2.1	Yes
**Methods**	8	Describe data sources and measurement/assessment for variables and comparability between groups.	Methods 2.3; Supplementary File S2	Yes
**Methods**	9	Describe measures used to address potential bias.	Methods 2.4	Yes
**Methods**	10	Explain how study size was determined.	Methods 2.2; Supplementary File S2, Section 6	Yes
**Methods**	11	Explain handling and categorization/scaling of quantitative variables.	Methods 2.3 and 2.5; Supplementary File S2, Tables S2.1–S2.2	Yes
**Methods**	12a	Describe statistical methods, including control of confounding.	Methods 2.5; Supplementary File S2, Section 9	Yes
**Methods**	12b	Describe subgroup, trend, or interaction analyses where performed.	Methods 2.5; Supplementary File S2, Section 9	Yes
**Methods**	12c	Explain how missing data were handled.	Methods 2.4; Supplementary File S2, Section 8.4	Yes
**Methods**	12d	Explain handling of matching in the analysis, if applicable.	Not applicable: controls were not matched.	N/A

Section	Item	STROBE requirement (condensed)	Where addressed in manuscript	Status
**Methods**	12e	Describe sensitivity analyses.	Methods 2.5; Supplementary File S2, Section 9.8	Yes
**Results**	13a	Report participant numbers at each stage from source population to analysis.	Results 3.1; Figure 1	Yes
**Results**	13b	Give reasons for exclusion/non-participation at each stage.	Results 3.1; Figure 1	Yes
**Results**	13c	Consider a flow diagram.	Figure 1	Yes
**Results**	14a	Report participant characteristics, exposure, and potential confounders separately for cases and controls.	Results 3.2; Table 1	Yes
**Results**	14b	Report missing data for variables of interest.	Results 3.1–3.2; Table 1/data-quality description	Yes
**Results**	15	Report exposure-category numbers or appropriate exposure summaries separately for cases and controls.	Results 3.2; Table 1	Yes
**Results**	16a	Report crude and adjusted estimates with precision and identify confounders included.	Results 3.3; Table 2; Figure 2	Yes
**Results**	16b	Report category boundaries when continuous variables are categorized.	Methods 2.3; Results 3.4; Table 3	Yes
**Results**	16c	Where relevant, translate relative effects into absolute risks over a meaningful period.	Not applicable to the fixed-ratio case–control analytical sample; source-population transfusion prevalence is reported separately.	N/A
**Results**	17	Report other analyses, including subgroup/trend/explanatory and sensitivity analyses.	Results 3.5–3.7; Table 4; Figure 3	Yes
**Discussion**	18	Summarize key results in relation to the study objectives.	Discussion 4.1	Yes
**Discussion**	19	Discuss limitations, potential bias/imprecision, and likely direction where possible.	Discussion 4.6	Yes
**Discussion**	20	Provide a cautious interpretation considering limitations and external evidence.	Discussion 4.1–4.6	Yes
**Discussion**	21	Discuss generalizability/external validity.	Discussion 4.4–4.6	Yes
**Other information**	22	State funding and the role of funders.	Back Matter: Funding	Yes

Reference: von Elm E, Altman DG, Egger M et al. The Strengthening the Reporting of Observational Studies in Epidemiology (STROBE) Statement: Guidelines for Reporting Observational Studies. Lancet. 2007;370:1453–1457. doi:10.1016/S0140-6736(07)61602-X. Checklist adapted for the present case–control report.
