# Supplementary material for: Predictors of Blood Transfusion Following Cesarean Delivery in Resource-Constrained Settings: The Impact of Out-of-Hours Surgery
Source: J Clin Med. 2026 Nov 6;15(21):8646. doi: 10.3390/jcm15218646 (PMC13764284; doi:10.3390/jcm15218646)
Supplement: Supplementary file 1 [file jcm-15-08646-s001.zip › Supplementary_File_S2_Protocol_and_Statistical_Analysis_Plan(1).pdf]

Supplementary File S2

Study Protocol and Prespecified Statistical Analysis Plan

Predictors of Blood Transfusion Following Cesarean Delivery in Resource-Constrained Settings: The Impact of Out-of-Hours Surgery

Cezara Mureșan; Marius Biriș; Răzvan Roșca; Andrei Pescaru; Cosmin Cîtu; Jasmina Chiriac; Denis Mihai Șerban; Răzvan Betea; Sebastian Ciurescu; Victor Bogdan Buciu

1. Rationale and Research Question

Perioperative red blood cell (RBC) transfusion after cesarean delivery is determined by maternal hematological reserve, obstetric hemorrhagic risk, and the organization of perioperative care. The study was designed to evaluate whether surgery performed outside routine working hours carries an independent transfusion association after accounting for major maternal, obstetric, placental, and preoperative hematological factors. The setting is a Romanian tertiary maternity hospital with a high cesarean burden and constrained out-of-hours resources. The protocol focuses on transfusion as an objective, routinely recorded outcome and uses postoperative/pre-transfusion hemoglobin only as a secondary explanatory variable because it occurs after the exposure.

Research question: Among women undergoing cesarean delivery with preoperative hemoglobin ≥10 g/dL and no preoperative coagulopathy, is out-of-hours surgery associated with higher odds of receiving ≥1 packed RBC unit intraoperatively or within 24 h compared with in-hours surgery?

2. Objectives and Hypotheses

Primary objective: The primary aim was to determine whether out-of-hours cesarean delivery is independently associated with increased odds of perioperative RBC transfusion compared with in-hours cesarean delivery.

Primary hypothesis: Out-of-hours cesarean delivery will remain associated with higher transfusion odds after adjustment for prespecified maternal, obstetric, placental, and preoperative hematological confounders.

Secondary objectives. The secondary aims were to identify other transfusion-associated factors; evaluate the contribution of previous cesarean burden, placenta previa, and suspected PAS; compare preoperative and postoperative/pre-transfusion hemoglobin and ΔHb; examine attenuation of the out-of-hours estimate after addition of postoperative/pre-transfusion hemoglobin; characterize transfusion timing and RBC-unit burden; assess peripartum hysterectomy and reintervention for hemorrhage; and describe the 1-minute Apgar score, including Apgar <7.

3. Study Design, Setting, and Source Population

The study is a retrospective, observational, single-center, unmatched case–control study nested within all cesarean deliveries performed at a tertiary maternity hospital in Romania between 1 January 2023 and 31 December 2025. The institution records approximately 3000 deliveries annually, approximately 75% of which are cesarean delivery. All variables are obtained from routinely maintained clinical and laboratory records; no study-specific intervention or investigation is performed.

The source population consists of all women who underwent cesarean delivery at the study institution during the study period. Eligibility is determined before case–control sampling and is applied identically to potential cases and controls.

4. Eligibility, Case Definition, Control Selection, and Exposure

4.1. Eligibility criteria

Inclusion criteria: Age ≥18 years; gestational age ≥24 weeks; cesarean delivery at the study institution; preoperative hemoglobin ≥10 g/dL obtained before any blood product; availability of postoperative hemoglobin or, when transfusion began intraoperatively, a pre-transfusion hemoglobin value; available preoperative PT, INR, and aPTT; determinable perioperative RBC transfusion status; and determinable in-hours/out-of-hours classification.

Exclusion criteria: Preoperative hemoglobin <10 g/dL; abnormal PT, INR, or aPTT according to institutional reference ranges or a known clinically relevant coagulopathy; cesarean delivery performed elsewhere followed by postpartum

transfer; missing essential hemoglobin, transfusion, or exposure data; or records insufficient to establish eligibility, exposure, or outcome. The hematological restrictions are intended to reduce transfusion primarily attributable to pre-existing anemia or coagulopathy and to create a more comparable preoperative baseline.

4.2. Cases and controls

Cases are all eligible women receiving ≥1 unit of packed RBCs intraoperatively or within the first 24 h after cesarean delivery. Controls are eligible women who received no RBC transfusion during the same perioperative interval. All eligible cases are retained. Controls are sampled without replacement at a 1:2 case-to-control ratio using computer-generated random sampling stratified by calendar year (2023, 2024, and 2025) with a reproducible seed (SET SEED = 2026). No matching is performed for exposure or clinical variables. Year stratification is used only to maintain temporal representation of the source population.

4.3. Primary exposure

The primary exposure is the institutionally recorded working-period classification of the cesarean delivery. In-hours procedures are those with an operative start between 08:00 and 15:00, Monday to Friday, excluding official public holidays. Out-of-hours procedures include all procedures starting outside this interval, including weekday evening/night duty periods, weekends, and public holidays. Classification uses the operative start time recorded in the institutional operative register and is applied independently of transfusion status. Elective/emergency status is not used because historical coding and indications are insufficiently standardized for reproducible retrospective classification.

5. Outcomes and Prespecified Variables

Primary outcome: Perioperative RBC transfusion, defined as receipt of ≥1 packed RBC unit intraoperatively or within 24 h after cesarean delivery.

Secondary maternal outcomes: Postoperative/pre-transfusion hemoglobin; ΔHb; transfusion timing (intraoperative or postoperative); number of RBC units; peripartum hysterectomy; and surgical reintervention for hemorrhage. ICU admission is not used because local ICU admission practice is not considered a reproducible severity endpoint. Neonatal status is described using the routinely recorded 1-minute Apgar score and Apgar <7; neonatal measures are not included as predictors in the maternal transfusion model.

Table S2.1. Variable definitions and coding

Variable	Definition	Coding/scale	Operational note
**Study_ID**	Unique pseudonymous record identifier	String/integer	No direct identifier retained
**Case_Status**	Perioperative RBC transfusion	0=control; 1=case	≥1 packed RBC unit intraoperatively or ≤24 h
**Out_of_Hours**	Primary exposure	0=in-hours; 1=out-of-hours	Weekday regular schedule vs duty period/weekend/public holiday
**Calendar_Year**	Year of cesarean	2023/2024/2025	Adjustment + sampling stratum
**Age_years**	Maternal age	Continuous	Also modeled per 5-year increase
**BMI_kg_m2**	Pre-pregnancy/available maternal BMI	Continuous	Also modeled per 5 kg/m² increase
**Parity**	Previous births	Integer	Continuous in main model
**GA_weeks**	Gestational age at delivery	Continuous	Weeks
**Multiple_Pregnancy**	Multiple gestation	0/1	Prespecified confounder
**Previous_CS_Count**	Exact prior cesarean count	Integer	Retained for trend/exploratory analysis
**Previous_CS_Category**	Uterine scar burden	0, 1, 2, ≥3	0 is reference category
**Placenta_Previa**	Placenta previa	0/1	Clinical/imaging record
**Suspected_PAS**	Suspected placenta accreta spectrum	0/1	Clinical/imaging suspicion; not uniformly histological

Variable	Definition	Coding/scale	Operational note
**Preeclampsia**	Preeclampsia	0/1	Clinical record
**Hb_Preop_g_dL**	Latest Hb before cesarean/blood product	Continuous	Eligibility requires ≥10 g/dL
**Hb_Post_PreTx_g_dL**	First postoperative Hb or last Hb before first intraoperative RBC unit	Continuous	Point-of-care blood-gas Hb allowed when applicable
**Delta_Hb_g_dL**	Hb preop − Hb post/pre-transfusion	Continuous	Complementary marker only
**Platelets_x10e9_L**	Preoperative platelet count	Continuous	Modeled per 50×10^9/L increase
**PT_sec**	Preoperative PT	Continuous	Eligibility check
**INR**	Preoperative INR	Continuous	Eligibility check
**aPTT_sec**	Preoperative aPTT	Continuous	Eligibility check
**Birth_Weight_g**	Neonatal birth weight	Continuous	Modeled per 500-g increase
**RBC_Units**	Packed RBC units administered	Integer	Cases only
**Transfusion_Timing**	Timing of first transfusion	Intraoperative/postoperative	Cases only
**Hysterectomy**	Peripartum hysterectomy	0/1	Secondary outcome
**Reintervention_Hemorrhage**	Surgical reintervention for hemorrhage	0/1	Secondary outcome
**Apgar_1min**	1-minute Apgar score	0–10	Secondary descriptive neonatal outcome
**Apgar_lt7**	1-minute Apgar <7	0/1	Derived from Apgar_1min

6. Study Size and Sampling Rationale

The analytical sample size is determined by the number of eligible transfusion cases occurring in the source population during the fixed 3-year period. All eligible cases are included. Two controls per case are selected because increasing the control-to-case ratio from 1:1 to 1:2 improves statistical efficiency while limiting unnecessary data abstraction; no post hoc case exclusion is permitted to achieve a target effect size. The study does not estimate transfusion incidence from the fixed-ratio analytical sample. Source-population counts may be used separately to describe the institutional transfusion prevalence.

No outcome-based power calculation is used to select the number of cases because the design includes all eligible cases from the prespecified period. Model complexity will be judged against the available case count, coefficient stability, confidence-interval width, and evidence of sparse-data problems, particularly for rare predictors such as suspected PAS.

7. Data Extraction and Quality Control

Two investigators independently extract, code, compile, and verify data using a predefined form and codebook. Discrepancies in eligibility, working-period exposure, laboratory values, transfusion status, obstetric variables, and neonatal data are resolved by review of the original source record and consensus. Each included woman receives a unique study code, and direct identifiers are removed before creation of the analytical dataset.

Data organization, coding, cleaning, and verification are performed in Microsoft Excel 2016 (Microsoft Corporation, Redmond, WA, USA), and statistical analyses are conducted in IBM SPSS Statistics for Windows, Version 26.0 (IBM Corp., Armonk, NY, USA). The analytical dataset is locked before execution of the prespecified primary model.

8. Bias Control and Missing Data

8.1. Selection bias

Selection bias is reduced by including all eligible cases, applying identical eligibility criteria before case/control classification, and randomly sampling controls from the same institution and calendar years as cases. Controls are selected without replacement and without matching on the primary exposure or clinical characteristics.

8.2. Information and classification bias

Prespecified operational definitions are used for exposure, outcome, laboratory variables, placental pathology, and uterine scar burden. Duplicate independent extraction is used to reduce transcription and coding error. Poorly standardized elective/emergency coding is deliberately excluded from the main analysis.

8.3. Confounding

Potential confounders are selected a priori on clinical grounds and include calendar year, maternal age, BMI, parity, gestational age, multiple pregnancy, previous cesarean category, placenta previa, suspected PAS, preeclampsia, preoperative hemoglobin, platelet count, and birth weight. Postoperative/pre-transfusion hemoglobin is not treated as a baseline confounder because it is measured after the exposure and may lie on the causal pathway.

8.4. Missing data

Records missing information required to determine eligibility, case/control status, baseline hemoglobin/coagulation status, or the primary exposure are excluded before sampling or analysis. Missingness in nonessential covariates is reported by group. Complete-case analysis is prespecified when missingness is ≤5%. If >5% of observations are missing for a model covariate and a missing-at-random mechanism is clinically plausible, multiple imputation by fully conditional specification is used, with complete-case results as the sensitivity analysis. Mean or median single-value imputation is prohibited.

9. Prespecified Statistical Analysis Plan

9.1. General principles

All tests are two-sided and statistical significance for the primary analysis is defined as $p<0.05$. Effect estimates are reported with 95% confidence intervals. Because the analytical case/control ratio is fixed by design, the principal effect measures are odds ratios (ORs) and adjusted odds ratios (aORs), and not risk ratios or absolute risks derived from the analytical sample.

9.2. Descriptive and univariable analyses

Continuous variables are assessed using histograms, Q-Q plots, skewness, and Shapiro–Wilk testing when appropriate and summarized as mean ± SD or median [IQR]. Categorical variables are summarized as n (%). Cases and controls are compared with independent-samples t tests, Mann–Whitney U tests, Pearson chi-square tests, or Fisher's exact tests as appropriate. Standardized mean differences may additionally describe group imbalance. Crude associations with case status are estimated by univariable binary logistic regression.

9.3. Primary analysis

The primary analysis is multivariable binary logistic regression with transfusion case status as the dependent variable and out-of-hours cesarean delivery as the exposure of interest. Prespecified covariates are calendar year, maternal age, BMI, parity, gestational age, multiple pregnancy, previous cesarean category, placenta previa, suspected PAS, preeclampsia, preoperative hemoglobin, platelet count, and birth weight. Covariates are retained according to the prespecified clinical model rather than selected solely by univariable p values. Age and BMI are expressed per 5-unit increase; platelets per 50×10^9/L; birth weight per 500 g; and hemoglobin per 1 g/dL. The unscarred uterus is the reference for previous cesarean categories.

Model diagnostics include inspection of sparse cells and unstable coefficients, correlation assessment, and variance inflation factors (VIFs) for multicollinearity. A year-stratified Mantel–Haenszel pooled OR for out-of-hours exposure and a test of heterogeneity across calendar years are used as a design-consistency check.

9.4. Hematological analyses

Preoperative hemoglobin, postoperative/pre-transfusion hemoglobin, and ΔHb are compared between cases and controls. Postoperative/pre-transfusion hemoglobin is also categorized as <7.0, 7.00–7.99, 8.00–8.99, 9.00–9.99, and ≥10.0 g/dL for descriptive comparison. ΔHb is not used as an isolated transfusion threshold.

9.5. Explanatory sequential model

A prespecified explanatory model adds postoperative/pre-transfusion hemoglobin to the complete primary covariate set. The purpose is to determine whether the out-of-hours coefficient is attenuated after accounting for the hematological state immediately preceding transfusion. Because this hemoglobin value is post-exposure, the model is interpreted as explanatory rather than as the principal causal/confounding model; attenuation is not considered proof of mediation.

9.6. Previous cesarean burden

Previous cesarean delivery is analyzed categorically as 0, 1, 2, and ≥3, with 0 as the reference. The exact previous cesarean count is retained for an exploratory linear trend assessment when model stability permits. Placenta previa and suspected PAS remain separate covariates because the effect of prior cesarean burden may be partly expressed through abnormal placentation.

9.7. Secondary outcomes

Among transfused cases, RBC-unit burden and transfusion timing are compared between in-hours and out-of-hours surgery. RBC units are summarized primarily as median [IQR] and compared using Mann–Whitney testing; negative-binomial modeling may be used only as an exploratory analysis if dispersion and sample size justify it. Hysterectomy and reintervention are compared using Fisher's exact tests when expected counts are small; adjusted models are attempted only if event counts are sufficient. The 1-minute Apgar score and Apgar <7 are secondary descriptive outcomes because the analytical sample is selected based on maternal transfusion status rather than neonatal outcome.

9.8. Sensitivity analyses

The adjusted out-of-hours association is repeated in three prespecified restricted samples: (1) excluding suspected PAS; (2) excluding both suspected PAS and placenta previa; and (3) excluding women whose first RBC transfusion began intraoperatively. These analyses assess whether the primary result is driven by major placental hemorrhagic pathology or by special measurement circumstances of intraoperative transfusion. A Firth-penalized logistic regression of the primary model, with profile penalized-likelihood confidence intervals, was added post hoc to assess sparse-data bias arising from the small number of controls with suspected PAS; it is reported as a post hoc analysis in the manuscript.

Table S2.2. Analysis matrix

Analysis	Outcome	Main comparison	Method	Report
**Primary**	Transfusion case status	Out-of-hours	Multivariable logistic regression	aOR, 95% CI, p
**Design consistency**	Transfusion case status	Out-of-hours stratified by year	Mantel-Haenszel	Pooled OR + heterogeneity p
**Hematology**	Hb post/pre-Tx; ΔHb	Case vs control	t/Mann–Whitney; χ^2 for Hb categories	Mean/median difference; p
**Explanatory**	Transfusion case status	Out-of-hours + post/pre-Tx Hb	Sequential logistic regression	Change in out-of-hours aOR
**Scar burden**	Transfusion case status	Previous CS 0/1/2/≥3	Logistic regression	OR/aOR; trend is stable
**Case-only burden**	RBC units	In-hours vs out-of-hours	Mann–Whitney; optional negative binomial	Median [IQR]; exploratory IRR
**Rare maternal outcomes**	Hysterectomy/reintervention	Case/control or in/out as specified	Fisher exact; adjusted only if feasible	n (%), p; OR if estimable
**Neonatal descriptive**	Apgar 1 min; <7	Relevant groups	Mann–Whitney/χ^2 or Fisher	Median [IQR], n (%)
**Sensitivity**	Transfusion case status	Out-of-hours	Same adjusted logistic model in	aOR, 95% CI, p

Analysis	Outcome	Main comparison	Method	Report
			restricted samples	

10. Ethics, Data Protection, and Confidentiality

The study was conducted in accordance with the 2024 revision of the World Medical Association Declaration of Helsinki and Regulation (EU) 2016/679 (General Data Protection Regulation). Ethics approval was granted by the Ethics Committee of the University of Medicine and Pharmacy "Victor Babeș" Timișoara, Approval No. 132583/2026. Informed consent was waived because of the retrospective nature of the study, in accordance with the ethics committee's decision. Data used for analysis are pseudonymized and stored with access restricted to authorized institutional investigators. No direct patient identifiers are included in analytical or supplementary datasets.

11. Reporting, Transparency, and Deviations

Reporting follows the STROBE recommendations for case–control studies. The completed checklist is provided as Supplementary File S1. The protocol and statistical analysis plan were finalized before the analytical dataset was locked and the prespecified analyses were performed. Any deviation from the prespecified eligibility criteria, exposure/outcome definitions, control-sampling procedure, primary covariate set, missing-data plan, or sensitivity analyses must be documented, dated, justified, and clearly identified as post hoc in the final report.

12. References

1. von Elm E, Altman DG, Egger M, Pocock SJ, Gøtzsche PC, Vandenbroucke JP; STROBE Initiative. The Strengthening the Reporting of Observational Studies in Epidemiology (STROBE) Statement: Guidelines for Reporting Observational Studies. Lancet. 2007;370:1453–1457. doi:10.1016/S0140-6736(07)61602-X.
2. World Medical Association. World Medical Association Declaration of Helsinki: Ethical Principles for Medical Research Involving Human Participants. JAMA. 2025;333:71–74. doi:10.1001/jama.2024.21972.
3. European Parliament; Council of the European Union. Regulation (EU) 2016/679 (General Data Protection Regulation). Off J Eur Union. 2016;L119:1–88.
4. Microsoft Corporation. Microsoft Excel 2016. Redmond, WA, USA: Microsoft Corporation; 2016.
5. IBM Corp. IBM SPSS Statistics for Windows, Version 26.0. Armonk, NY, USA: IBM Corp.; 2019.
